# Halogen-Substituted Derivatives of *Dictyostelium* Differentiation-Inducing Factor-1 Suppress Serum-Induced Cell Migration of Human Breast Cancer MDA-MB-231 Cells in Vitro

**DOI:** 10.3390/biom9070256

**Published:** 2019-06-28

**Authors:** Kyoko Totsuka, Yuka Makioka, Kyoichi Iizumi, Katsunori Takahashi, Yoshiteru Oshima, Haruhisa Kikuchi, Yuzuru Kubohara

**Affiliations:** 1 Laboratory of Health and Life Science, Graduate School of Health and Sports Science, Juntendo University, Inzai, Chiba 270-1695, Japan; 2 Laboratory of Health and Life Science, Department of Health Science, Faculty of Health and Sports Science, Juntendo University, Inzai, Chiba 270-1695, Japan; 3 Department of Medical Technology, Faculty of Health Science, Gunma Paz College, Takasaki 370-0006, Japan; 4 Laboratory of Natural Product Chemistry, Graduate School of Pharmaceutical Sciences, Tohoku University, Aoba-ku, Sendai 980-8578, Japan

**Keywords:** triple-negative breast cancer, metastasis, cell migration, anticancer drug, *Dictyostelium discoideum*, DIF

## Abstract

Triple-negative breast cancer (TNBC) is highly proliferative and metastatic, and because it lacks three major molecular targets for chemotherapy (estrogen receptor, progesterone receptor, and human epidermal receptor 2), it is extremely refractory. Differentiation-inducing factor 1 (DIF-1) and DIF-3, which are chlorinated alkylphenones, are lead anticancer compounds found in the cellular slime mold *Dictyostelium discoideum*. Here, we examined the in vitro effects of DIF-1, DIF-3, and 25 DIF derivatives on cell proliferation and serum-induced cell migration in human MDA-MB-231 cells, a model TNBC cell line. We found that Br-DIF-1, a chlorine-to-bromine-substituted derivative of DIF-1, strongly suppressed cell migration (IC_50_, 3.8 μM) with negligible effects on cell proliferation (IC_50_, >20 μM). We then synthesized 18 derivatives of Br-DIF-1 and examined the in vitro effects of these derivatives on cell proliferation and serum-induced cell migration in MDA-MB-231 cells. Among the derivatives, Br-DIF-1(+1), Br-DIF-1(+2), and Br-DIF-3(+2) exhibited strong anti-cell migration activities with IC_50_ values of 1.5, 1.0, and 3.1 μM, respectively, without affecting cell proliferation (IC_50_, >20 μM). These results suggest that these Br-DIF derivatives are good lead compounds for the development of anti-metastatic drugs against TNBC.

## 1. Introduction

Breast cancer is the most common cancer affecting women, accounting for 25% of all cancers in women [1,2]. Breast cancers are categorized according to histopathological type, grade, tumor stage, and receptor protein and gene expression [2,3,4,5]. For example, four subtypes of breast cancer categorized by receptor status are i) luminal A (estrogen receptor (ER) positive, progesterone receptor (PR) positive, and human epidermal receptor 2 (HER2) negative), ii) luminal B (ER-positive and/or PR-positive, and HER2-positive), iii) HER2-enriched (ER-negative, PR-negative, and HER2-positive), and iv) triple-negative (ER-negative, PR-negative, and HER2 negative) breast cancer [2]. Of these four subtypes, triple-negative breast cancer (TNBC) accounts for 15–20% of all breast cancers. Because TNBC is highly proliferative and metastatic and because molecular targeted therapies available for the treatment of other breast cancers are unavailable, TNBC is extremely refractory [4,6,7,8,9]. Therefore, innovative breast cancer treatments, including the development of novel anticancer and anti-metastasis drugs, are needed.

**Figure 1:**
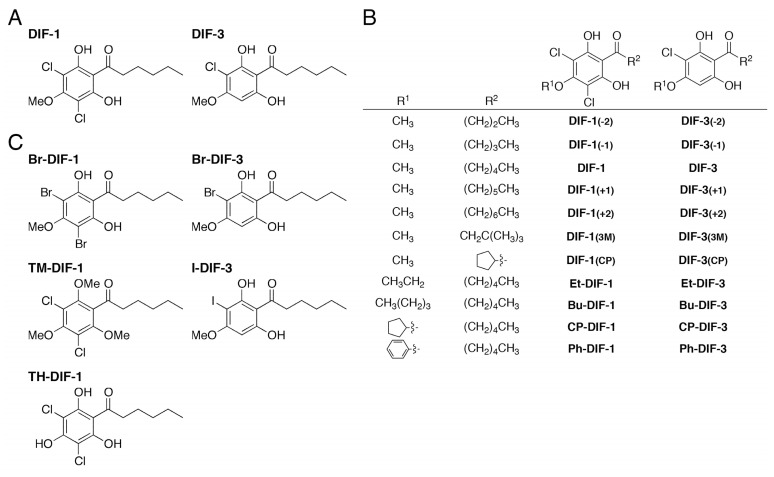
(**A**) Chemical structures of DIF1 (differentiation inducing factor 1) and DIF-3. (**B**,**C**) Chemical structures of the derivatives of DIF-1 and DIF-3 used in the experiment shown in Figure 3.

Differentiation-inducing factor 1 (DIF-1) is a chlorinated polyketide (Figure 1A) that was originally isolated as an inducer of stalk cell differentiation in the cellular slime mold *Dictyostelium discoideum* [10]. DIF-3 (Figure 1A) is a metabolite of DIF-1 and has no remarkable biological activity in *D. discoideum* [11,12]. However, we have shown that DIF-1, DIF-3, and several of their derivatives have anti-proliferative and anti-metastatic activities in mammalian tumor cells in vitro and in vivo [13,14,15,16,17,18,19,20,21,22,23,24,25,26]. Recently, we examined the effects of DIF-1, DIF-3, and 25 DIF derivatives (Figure 1B, C) on cell proliferation and lysophosphatidic acid-induced cell migration (LICM) in murine osteosarcoma LM8 cells (an in vitro model system of metastasis) by using a Boyden chamber, and we found that while several of the DIF derivatives strongly suppressed LICM, their effects on cell proliferation varied; for example, Bu-DIF-3 (Figure 1B) strongly suppressed both cell proliferation and LICM, whereas Br-DIF-1 (Figure 1C) strongly suppressed LICM but not cell proliferation [21].

**Figure 2:**
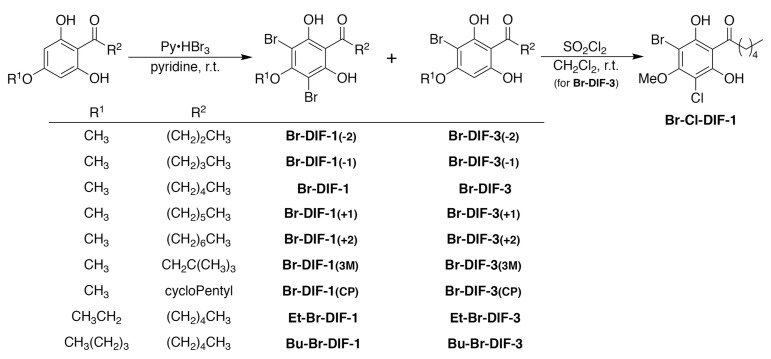
Synthetic routes and chemical structures of Br-DIF-1 and its derivatives.

In the present study, to screen for strong anti-metastatic compounds against TNBC, we examined the in vitro effects of DIF derivatives on serum-induced cell migration (SICM) in human MDA-MB-231 cells, a model TNBC cell line. We found that Br-DIF-1 strongly suppressed SICM without markedly affecting cell proliferation. We then synthesized 18 derivatives of Br-DIF-1 (Figure 2) and examined the effects of the derivatives on SICM in MDA-MB-231 cells, and we found that several of the novel derivatives suppressed SICM more strongly than did Br-DIF-1 without markedly affecting cell proliferation. These results suggest that derivatives of Br-DIF-1 are good lead compounds for the development of anti-metastatic drugs against TNBC.

## 2. Materials and Methods 

### 2.1. Cells and Reagents

Human MDA-MB-231 cells were obtained from ATCC (Manassas, VA, US). Cells were maintained at 37 °C (5% CO_2_ in air) in DMEM-FBS (Dulbecco’s modified Eagle’s medium containing 4500 mg/mL of glucose (Wako Pure Chemical Industries, Osaka, Japan) supplemented with 100 units/mL of penicillin, 100 μg/mL of streptomycin, and 10% (*v*/*v*) fetal bovine serum (FBS)). 

### 2.2. Synthesis of DIF Derivatives

DIF-1, DIF-3, and their derivatives (Figure 1) were synthesized as previously described [19]. We then synthesized Br-DIF derivatives as follows (Figure 2): 1-(2,6-dihydroxy-4-methoxyphenyl)hexan-1-one (145 mg, 0.609 mmol) was dissolved in pyridine (4 mL). Then, pyridinium tribromide (350 mg, 1.10 mmol) was added to the solution at room temperature. After stirring for 1 h, the reaction mixture was poured into water (10 mL) and extracted with ethyl acetate (20 mL) three times. The organic layer was washed with brine, dried over sodium sulfate, and concentrated *in vacuo*. The residue was chromatographed over silica gel and eluted with hexane-ethyl acetate (4:1) to afford Br-DIF-1 (149 mg, 0.375 mmol) and Br-DIF-3 (54 mg, 0.170 mmol). Other Br-DIFs were synthesized from 1-(2,6-dihydroxy-4-alkoxyphenyl)hexan-1-one or 1-(2,6-dihydroxy-4-methoxyphenyl)alkan-1-one by following a similar procedure.

### 2.3. Data for Br-DIF Derivatives

*Br-DIF-1*: Yellow amorphous solid; HREIMS *m*/*z* 393.9428 [M]^+^ (393.9415 calculated for C_13_H_16_O_4_^79^Br_2_); NMR data are reported in our previous paper [19].

*Br-DIF-3*: Colorless amorphous solid; HREIMS *m*/*z* 316.0296 [M]^+^ (316.0310 calculated for C_13_H_17_O_4_^79^Br); NMR data are reported in our previous paper [19].

*Br-DIF-1(–2)*: Yellow amorphous solid; HREIMS *m*/*z* 365.9088 [M]^+^ (365.9102 calculated for C_11_H_12_O_4_^79^Br_2_).

*Br-DIF-3(–2)*: Colorless amorphous solid; HREIMS *m*/*z* 288.0004 [M]^+^ (287.9997 calculated for C_11_H_13_O_4_^79^Br).

*Br-DIF-1(–1)*: Yellow amorphous solid; HREIMS *m*/*z* 379.9271 [M]^+^ (379.9259 calculated for C_12_H_14_O_4_^79^Br_2_).

*Br-DIF-3(–1)*: Colorless amorphous solid; HREIMS *m*/*z* 302.0159 [M]^+^ (302.0154 calculated for C_12_H_15_O_4_^79^Br).

*Br-DIF-1(+1)*: Yellow amorphous solid; HREIMS *m*/*z* 407.9597 [M]^+^ (407.9572 calculated for C_14_H_18_O_4_^79^Br_2_).

*Br-DIF-3(+1)*: Colorless amorphous solid; HREIMS *m*/*z* 330.0480 [M]^+^ (330.0467 calculated for C_14_H_19_O_4_^79^Br).

*Br-DIF-1(+2)*: Yellow amorphous solid; HREIMS *m*/*z* 421.9734 [M]^+^ (421.9728 calculated for C_15_H_20_O_4_^79^Br_2_).

*Br-DIF-3(+2)*: Colorless amorphous solid; HREIMS *m*/*z* 344.0641 [M]^+^ (344.0623 calculated for C_15_H_21_O_4_^79^Br).

*Br-DIF-1(3M)*: Yellow amorphous solid; HREIMS *m*/*z* 393.9420 [M]^+^ (393.9415 calculated for C_13_H_16_O_4_^79^Br_2_).

*Br-DIF-3(3M)*: Colorless amorphous solid; HREIMS *m*/*z* 316.0301 [M]^+^ (316.0310 calculated for C_13_H_17_O_4_^79^Br).

*Br-DIF-1(CP)*: Yellow amorphous solid; HREIMS *m*/*z* 391.9276 [M]^+^ (391.9259 calculated for C_13_H_14_O_4_^79^Br_2_).

*Br-DIF-3(CP)*: Colorless amorphous solid; HREIMS *m*/*z* 314.0144 [M]^+^ (314.0154 calculated for C_13_H_15_O_4_^79^Br).

*Et-Br-DIF-1*: Yellow amorphous solid; HREIMS *m*/*z* 407.9578 [M]^+^ (407.9572 calculated for C_14_H_18_O_4_^79^Br_2_).

*Et-Br-DIF-3*: Colorless amorphous solid; HREIMS *m*/*z* 330.0478 [M]^+^ (330.0467 calculated for C_14_H_19_O_4_^79^Br).

*Bu-Br-DIF-1*: Yellow amorphous solid; HREIMS *m*/*z* 435.9880 [M]^+^ (435.9885 calculated for C_16_H_22_O_4_^79^Br_2_).

*Bu-Br-DIF-3*: Colorless amorphous solid; HREIMS *m*/*z* 358.0782 [M]^+^ (358.0780 calculated for C_16_H_23_O_4_^79^Br).

*Br-Cl-DIF-1*: Yellow amorphous solid; HREIMS *m*/*z* 349.9938 [M]^+^ (349.9920 calculated for C_13_H_16_O_4_^79^Br^35^Cl).

### 2.4. Cell migration Assay

**Figure 3:**
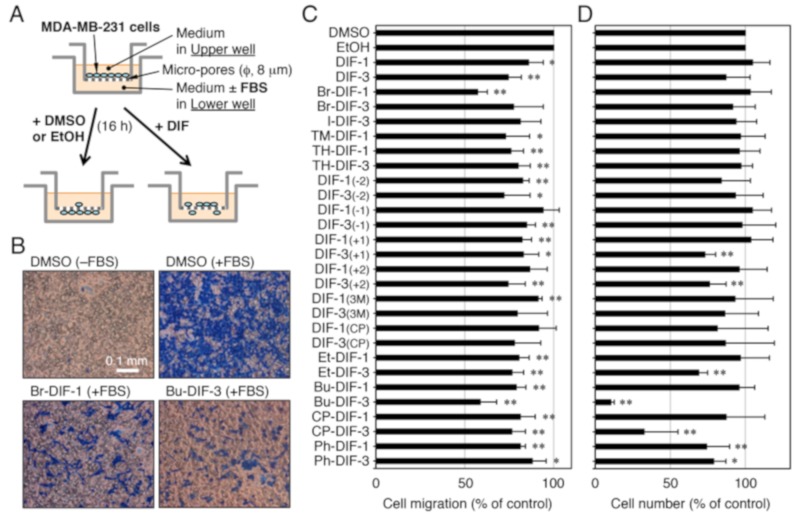
(**A**) Scheme for the in vitro cell migration assay using Trans-wells. MDA-MB-231 cells were put in the upper well (5 × 10^4^ cells/well), which has micro-pores running through the bottom of the well. The upper well was set on the lower well (with or without 10% FBS) and vehicle (0.1% DMSO or EtOH) or one of the DIFs was added to both the upper and lower wells. After incubation for 16 h at 37 °C, serum-induced cell migration (SICM) was assessed. (**B**) Representative photographs of migrated cells in Trans-wells. After incubation for 16 h at 37 °C with 0.1% DMSO or 10 μM of Br-DIF-1 or Bu-DIF-3 (in the upper and lower wells) in the presence or absence of 10% FBS (in the lower well). The cells that had migrated to the lower surface of the upper well were stained and observed microscopically. (**C**) Effects of DIF derivatives on SICM in MDA-MB-231 cells in Trans-wells. Cells were incubated for 16 h at 37 °C in the presence of vehicle (0.1% DMSO or EtOH) or 5 μM of the indicated DIF derivatives (in the upper and lower wells) and 10% FBS (in the lower well). SICM was determined relative to control and means and SDs (bars) were determined from four independent experiments. *, *P* < 0.05; **, *P* < 0.01 versus vehicle control. (**D**) Effects of DIF derivatives on cell proliferation in MDA-MB-231 cells. Cells were incubated for 3 days at 37 °C in the presence of vehicle (0.1% DMSO or EtOH) or 10 μM of the indicated DIF derivatives, and cell number was determined relative to control. Means and SDs (bars) were determined from four independent experiments. *, *P* < 0.05; **, *P* < 0.01 versus vehicle control.

Cell migration was assessed in vitro by using Trans-wells (#3422; Corning, New York, NY), as shown in Figure 3A. Trans-wells consist of two wells that fit inside one another, with the bottom portion of the upper well being a polycarbonate membrane containing micro-pores (pore size, 8 μm). MDA-MB-231 cells in DMEM-BSA (DMEM containing 4500 mg/mL of glucose supplemented with 100 units/mL of penicillin, 100 μg/mL of streptomycin, and 0.1% (*w*/*v*) bovine serum albumin) were added to the upper wells (5 × 10^4^ cells/0.3 mL/upper well), while the lower wells were filled with 0.5 mL of DMEM-BSA (–FBS) or DMEM-FBS (+FBS); the media in both the upper and lower wells contained 0.1% (*v*/*v*) vehicle (DMSO or EtOH) or 1–10 μM of a DIF derivative. The upper wells were set on the lower wells, and then both wells were incubated for 16 h at 37 °C (5% CO_2_ in air). After incubation, the cells in the upper wells were washed twice with phosphate-buffered saline, fixed for 3 min with methanol, and stained for 15 min with Victoria Blue solution (Nissui Seiyaku, Tokyo, Japan). After washing two more times with phosphate-buffered saline, the cells remaining in the upper well (i.e., those that did not migrate) were removed with cotton swabs, and the cells that had migrated to the lower well were counted by microscopic observation; four microscopic fields were examined per well and migration was assessed as the percentage of cells that had migrated toward the FBS relative to control.

To determine the 50% inhibitory concentration (IC_50_) of each DIF derivative versus cell migration, the cell migration assay was performed in the presence of a range of concentrations of DIF derivatives, and IC_50_ values were determined from the dose–response curves.

### 2.5. Cell Proliferation Assay

MDA-MB-231 cells (1 × 10^4^ cells/mL/well) were incubated for 3 days at 37 °C (5% CO_2_ in air) in 12-well plates with each well filled with 1 mL of DMEM-FBS. After the incubation medium was removed, the cells were washed with 1 mL of phosphate-buffered saline (pH 7.4) and incubated with 1 mL of fresh DMEM-FBS containing 5% (*v*/*v*) of the cell number indicator Alamar blue (Wako Pure Chemical Industries) until the color of the medium had changed. Relative cell number was determined by measuring absorbance at 570 nm (reference at 595 nm), as described previously [16,19].

To determine the 50% inhibitory concentration (IC_50_) of each DIF derivative versus cell proliferation, the cell proliferation assay was performed in the presence of a range of concentrations of DIF derivatives, and IC_50_ values were determined from the dose–response curves.

### 2.6. Statistical Analysis

Statistical analyses were performed by using Welch’s *t*-test (two-tailed); a difference was considered significant when the *P* value was less than 0.05.

## 3. Results

### 3.1. Effects of DIF Derivatives on Cell Migration and Cell Proliferation in MDA-MB-231 Cells

To investigate the structure–activity relationship of the DIF derivatives, we first examined the effects of the DIF derivatives (shown in Figure 1) on SICM in MDA-MB-231 cells in vitro (Figure 3A–C). Among the DIF derivatives examined, Br-DIF-1 and Bu-DIF-3 at 5 μM most strongly suppressed SICM (Figure 3B,C).

We then examined the effects of the DIF derivatives on cell proliferation in the MDA-MB-231 cells (Figure 3D). Bu-DIF-3 most strongly suppressed cell proliferation, whereas Br-DIF-1 had no significant effect.

### 3.2. Comparison of the Biological Activities of Br-DIF-1 and Bu-DIF-3

**Figure 4:**
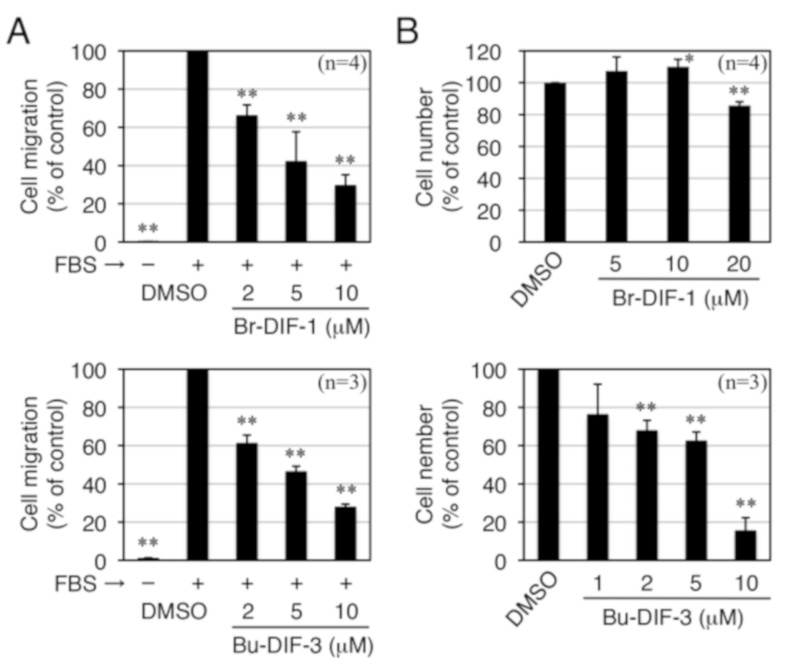
(**A**) Effects of Br-DIF-1 and Bu-DIF-3 on serum-induced cell migration in MDA-MB-231 cells in Trans-wells. Cells were incubated for 16 h at 37 °C in the presence of vehicle (0.1% DMSO) or the indicated concentrations of Br-DIF-1 or Bu-DIF-3 (in the upper and lower wells) and 0.1% BSA (–FBS) or 10% FBS (+FBS) (in the lower well). Serum-induced cell migration (SICM) was determined relative to control and means and SDs (bars) were determined from three or four independent experiments. **, *P* < 0.01 versus vehicle control. (**B**) Effects of Br-DIF-1 and Bu-DIF-3 on cell proliferation in MDA-MB-231 cells. Cells were incubated for 3 days at 37 °C in the presence of vehicle (0.1% DMSO) or the indicated concentrations of Br-DIF-1 and Bu-DIF-3, and cell number was assessed relative to control. Means and SDs (bars) were determined from three or four independent experiments. *, *P* < 0.05; **, *P* < 0.01 versus vehicle control.

To further assess the biological activities of Br-DIF-1 and Bu-DIF-3, IC_50_ values relative to SICM and cell proliferation were determined in MDA-MB-231 cells and compared with those relative to LICM and cell proliferation in mouse osteosarcoma LM8 cells and relative to cell proliferation in mouse 3T3-L1 fibroblast cells, which are a model non-transformed cell line (Figure 4, Table 1). Bu-DIF-3 strongly suppressed both cell migration and cell proliferation in all of the cell lines examined (Table 1), with IC_50_ values at the micromolar level. Similarly, Br-DIF-1 also showed strong anti-cell migration activity in both MDA-MB-231 and LM8 cells with IC_50_ values of 3.8 and 5.5 μM, respectively; however, its anti-proliferative activity was weak in MDA-MB-231, LM8, and 3T3-L1 cells (Table 1). Therefore, in subsequent experiments, we focused on Br-DIF-1 and its derivatives because we considered them to have the most potential for development as analytical reagents to investigate cancer cell migration or as anti-metastatic drugs.

### 3.3. Synthesis of Br-DIF-1 Derivatives and Structure–Activity Relationship Analysis

**Figure 5:**
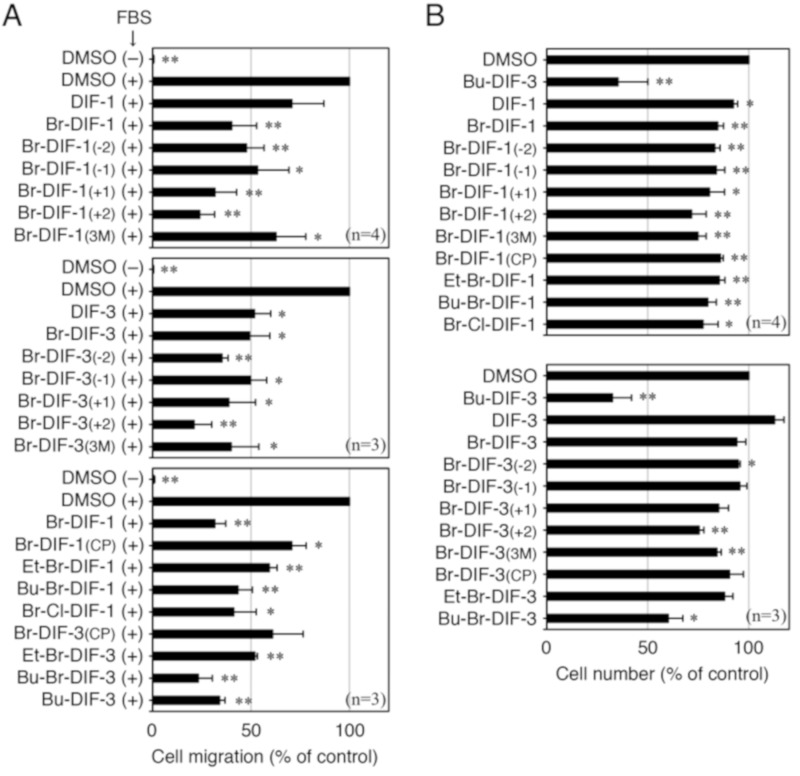
(**A**) Effects of Br-DIF-1 and Bu-DIF-3 on serum-induced cell migration in MDA-MB-231 cells in Trans-wells. Cells were incubated for 16 h at 37 °C in the presence of vehicle (0.1% DMSO) or 10 μM of the indicated DIF derivatives (in the upper and lower wells) and 0.1% BSA (–FBS) or 10% FBS (+FBS) (in the lower well). Serum-induced cell migration (SICM) was determined relative to control and means and SDs (bars) were determined from three or four independent experiments. **, *P* <0.01 versus vehicle control. (**B**) Effects of Br-DIF-1 and Bu-DIF-3 on cell proliferation in MDA-MB-231 cells. Cells were incubated for 3 days at 37 °C in the presence of vehicle (0.1% DMSO or EtOH) or 10 μM of the indicated DIF derivatives, and cell number was determined relative to control. Means and SDs (bars) were determined from three or four independent experiments. *, *P* < 0.05; **, *P* < 0.01 versus vehicle control.

Next, to screen for anti-metastatic seed compounds that are more effective than Br-DIF-1, we synthesized several derivatives of Br-DIF-1 (Figure 2) and examined their effects on SICM and cell proliferation in MDA-MB-231 cells (Figure 5). All of the novel Br-DIF derivatives at 10 μM significantly suppressed SICM, and most of them significantly suppressed cell proliferation in MDA-MB-231 cells.

**Figure 6:**
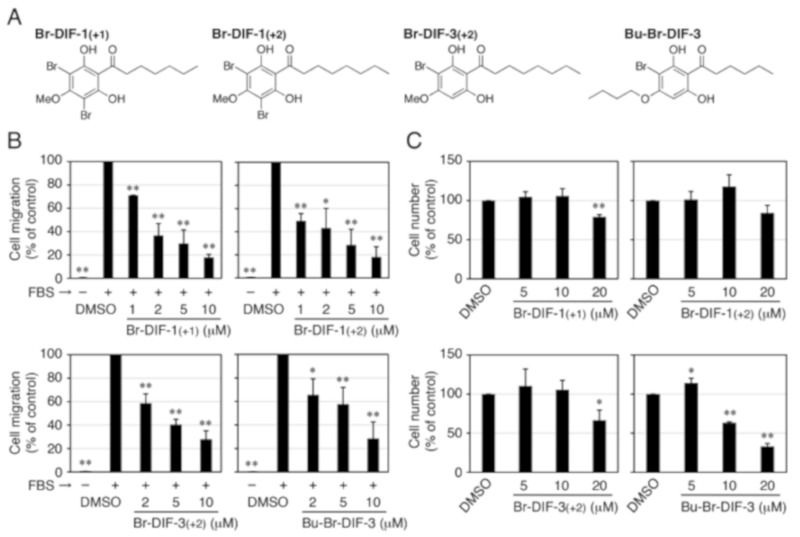
(**A**) Chemical structure of the Br-DIF derivatives that we further examined. (**B**) Effects of the Br-DIF derivatives on serum-induced cell migration (SICM) in MDA-MB-231 cells in Trans-wells. Cells were incubated for 16 h at 37 °C in the presence of vehicle (0.1% DMSO) or the indicated concentrations of Br-DIF derivatives (in the upper and lower wells) and 0.1% BSA (–FBS) or 10% FBS (+FBS) (in the lower well). SICM was determined relative to control and means and SDs (bars) were determined from four independent experiments. *, *P* < 0.05; **, *P* < 0.01 versus vehicle control. (**B**) Effects of Br-DIF derivatives on cell proliferation in MDA-MB-231 cells. Cells were incubated for 3 days at 37 °C in the presence of vehicle (0.1% DMSO) or the indicated concentrations of Br-DIF derivatives, and cell number was determined relative to control. Means and SDs (bars) were determined from four independent experiments. *, *P* < 0.05; **, *P* < 0.01 versus vehicle control.

Four of the derivatives—Br-DIF-1(+1), Br-DIF-1(+2), Br-DIF-3(+2), and Bu-Br-DIF-3 (Figure 6A)—had anti-SICM activity equal to or stronger than that of Br-DIF-1. We therefore assessed the IC_50_ values of these derivatives relative to SICM and cell proliferation in MDA-MB-231 cells (Figure 6B, Table 2). The IC_50_ values of Br-DIF-1(+1), Br-DIF-1(+2), and Br-DIF-3(+2) versus SICM were 1.5, 1.0, and 3.1 μM, respectively, which were lower than that of Br-DIF-1 (3.8 μM). Note that the IC_50_ values of the three Br-DIF derivatives relative to cell proliferation were more than 20 μM. The IC_50_ values of Bu-Br-DIF-3 relative to SICM and cell proliferation were 4.7 and 13.6 μM, respectively.

## 4. Discussion

Metastasis is one of the most common causes of death in breast cancer patients [27,28]. The sites of metastasis of breast cancer include the lung, brain, and liver, but the most common site is bone; breast cancer patients with bone metastases have a poor prognosis [29,30,31,32]. Because TNBC is highly proliferative and metastatic and because molecular targeted therapies available for the treatment of other breast cancers are unavailable, TNBC is extremely refractory [4,6,7,8,9]. Although investigations have been conducted to find novel chemotherapies for the treatment of TNBC [2,4,33,34,35], new approaches to improve the current situation are still urgently needed.

In the present study, we found that Bu-DIF-3 strongly suppressed both SICM and cell proliferation in MDA-MB-231 cells (Table 1). We also have shown previously that Bu-DIF-3 suppresses cell proliferation and LICM in mouse osteosarcoma LM8 cells in vitro [21] (Table 1), and that Bu-DIF-3 suppresses serum-dependent cell migration (wound healing) in LM8 cells in vitro [36]. These results suggest that Bu-DIF-3 is good a lead compound for the development of anticancer agents that suppress both cancer cell proliferation and metastasis, including TNBC. However, given that Bu-DIF-3 suppresses cell proliferation in 3T3-L1 cells (a model of non-transformed cells) to almost the same extent as in cancer cells [21] (Table 1), it may induce side effects such as diarrhea and hair loss in vivo. In addition, anti-proliferative drugs usually inhibit the proliferation of both normal and cancer cells, which limits the dose that can be used therapeutically. Therefore, if a drug that specifically inhibits metastasis can be developed, an effective cancer treatment could be realized in combination with existing anticancer drugs. We therefore screened derivatives of Br-DIF-1, which we found to inhibit cell migration but not proliferation, to find potential lead compounds for development as anti-metastatic drugs. Among the Br-DIF-1 derivatives examined, we found that Br-DIF-1(+1), Br-DIF-1(+2), and Br-DIF-3(+2) strongly suppressed SICM, but not cell proliferation, in MDA-MB-231 cells in vitro (Table 2). These results indicate that compounds with longer alkyl chains at the acyl group (e.g., Br-DIF-1(+1) and Br-DIF-1(+2)) have greater anti-cell migration activities than those with shorter alkyl chains. These results also suggest that Br-DIF derivatives could be useful tools for the analysis of cancer cell migration in vitro and good lead compounds for the development of anti-metastatic agents for use in vivo.

## 5. Conclusions

We examined the in vitro effects of *Dictyostelium* DIF-1 (a chlorinated polyketide) and its derivatives on SICM of human MDA-MB-231 cells, a model tripe negative breast cancer cell line. We found here that Br-DIF-1 (a chlorine-to-bromine substituted derivative of DIF-1) and several of its derivatives suppressed SICM strongly suppressed SICM without markedly affecting cell proliferation. The present results suggest that Br-DIF derivatives are promising lead compounds for the development of anti-metastatic drugs against TNBC.

## Figures and Tables

**Table 1:** IC_50_ values of DIF derivatives versus cell migration and cell proliferation in various cell lines.

	IC_50_ (μM) vs. Cell Migration		IC_50_ (μM) vs. Cell Proliferation		
Compound	MDA-MB-231 ^1^	LM8 ^2^	MDA-MB-231 ^1^	LM8 ^2^	3T3-L1 ^2^
DIF-1	>10	8.5	>20	18.2	>20
DIF-3	>10	10.2	>20	15.5	>20
Br-DIF-1	3.8	5.5	>20	18.5	>20
Bu-DIF-3	3.9	4.2	6.0	2.0	4.3

^1^ Data from the present study. ^2^ Data from our previous study [21], where the effects of DIFs on cell proliferation and lysophosphatidic acid-induced cell migration of murine osteosarcoma LM8 cells were assessed by using a Boyden chamber.

**Table 2:** IC_50_ values of Br-DIF derivatives versus serum-induced cell migration and cell proliferation in MDA-MB-231 cells.

Compound	IC_50_ (μM) vs. Cell Migration	IC_50_ (μM) vs. Cell Proliferation
Br-DIF-1	3.8	>20
Br-DIF-1(+1)	1.5	>20
Br-DIF-1(+2)	1.0	>20
Br-DIF-3(+2)	3.1	>20
Bu-Br-DIF-3	4.7	13.6
